# An accurate description of *Aspergillus niger* organic acid batch fermentation through dynamic metabolic modelling

**DOI:** 10.1186/s13068-017-0950-6

**Published:** 2017-11-09

**Authors:** Daniel J. Upton, Simon J. McQueen-Mason, A. Jamie Wood

**Affiliations:** 1 Department of Biology, University of York, Wentworth Way, York, YO10 5DD UK; 2 Department of Mathematics, University of York, Heslington, York, YO10 5DD UK

**Keywords:** *Aspergillus niger*, Citric acid, dFBA, Metabolic modelling, Polyphosphate

## Abstract

**Background:**

*Aspergillus niger* fermentation has provided the chief source of industrial citric acid for over 50 years. Traditional strain development of this organism was achieved through random mutagenesis, but advances in genomics have enabled the development of genome-scale metabolic modelling that can be used to make predictive improvements in fermentation performance. The parent citric acid-producing strain of *A. niger*, ATCC 1015, has been described previously by a genome-scale metabolic model that encapsulates its response to ambient pH. Here, we report the development of a novel double optimisation modelling approach that generates time-dependent citric acid fermentation using dynamic flux balance analysis.

**Results:**

The output from this model shows a good match with empirical fermentation data. Our studies suggest that citric acid production commences upon a switch to phosphate-limited growth and this is validated by fitting to empirical data, which confirms the diauxic growth behaviour and the role of phosphate storage as polyphosphate.

**Conclusions:**

The calibrated time-course model reflects observed metabolic events and generates reliable in silico data for industrially relevant fermentative time series, and for the behaviour of engineered strains suggesting that our approach can be used as a powerful tool for predictive metabolic engineering.

**Electronic supplementary material:**

The online version of this article (10.1186/s13068-017-0950-6) contains supplementary material, which is available to authorized users.

## Background

Due to its natural ability to secrete organic acids and proteins, the filamentous fungus *Aspergillus niger* is an established organism for the industrial production of citric acid and enzymes. *A. niger* is metabolically highly versatile, a feature that has made it useful for a wide range of biotechnological biotransformations [1]. *A. niger* also produces a wide range of secondary metabolites, with over 100 reported to date [2]. *A. niger* is a saprotroph and its natural habitat is soil, although it can be found in wide-ranging habitats, such as rotting fruit, plant debris, and indoor environments. This fast-growing fungus is both acid- and thermo-tolerant, able to grow in the pH range 1.4–9.8 and in the temperature range 6–47 °C [3]. This versatility and its ease of culture has helped it become an established industrial organism. Its haploid genome is around 35 Mb in size with eight chromosomes which contain about 12,000 genes, 57% of which have functional assignments [4]. Aspergilli are an important and diverse group, which in addition to *A. niger*, include well-studied species such as the model genetic organism *A. nidulans*, the pathogen *A. fumigatus* and the domesticated *A. oryzae*. Full genome sequences are currently available for 18 species of the Aspergilli group [5] and some of these have been subject to extensive systems biology studies [6].

With global production of 2 million tonnes a year, citric acid is an industrial chemical with many applications [7]. Its main use is in the food and drinks industry, but is also used in cleaning agents, pharmaceuticals, animal feed, and metal cleaning [8]. Industries using *A. niger* fermentation are dependent on sucrose-based feedstocks, but with rising costs and increasing concerns over food security, a switch to more sustainable and lower cost feedstocks is desirable [9]. *A. niger* can assimilate a wide range of carbon sources, and therefore has great potential for exploiting underused resource streams such as pentose sugars from lignocellulose.

The best industrial strains are capable of producing over 70% of the theoretical yield of citric acid [10]. Such strains have been developed over many decades by time-consuming random mutagenesis. The genotype of resulting strains remains unknown, and random mutagenesis can lead to genetic instability of developed strains. Rational engineering of *A. niger* is now feasible, particularly with advances in genomics over recent years that have paved the way for genome-scale metabolic modelling [5, 11]. Industrially, *A. niger* is utilised via large-scale batch fermentations rather than continuous culture methods, typically in reactors in excess of 100,000 L [12]. In order for genome-scale models to accurately capture the behaviour of these cultures, techniques which model the batch growth, rather than simple chemostat-like cultures, are required.

The genome of the parent citric acid-producing strain of *A. niger*, ATCC 1015, has been sequenced [4]. This enabled development of the genome-scale metabolic model for *A. niger*, *i*MA871, which reflects ATCC 1015 metabolism [13]. The model was further developed to reflect the well-known behaviour of *A. niger* to acidify its surroundings in response to ambient pH [14]. This was achieved by incorporating acid-dissociation reactions for seven organic acids reportedly secreted by *A. niger*. Each reaction gives the number of protons released by a particular acid as a function of ambient pH. Citric acid production was modelled statically using flux balance analysis (FBA). The objective function was either set to proton production at a fixed growth rate or proton production was incorporated into the biomass equation. The nature of organic acid production in response to ambient pH is, however, a dynamic one, with acid-dissociation reactions changing as protons are produced.

In this article, we further develop the *A. niger* metabolic model to take into account the dynamic nature of organic acid production. By designing a novel modelling approach that employs dynamic flux balance analysis (dFBA), we demonstrate a model that gives time-course fermentative series of citric acid production. We validate the new model by fitting to empirical data from ATCC 1015 citric acid fermentations, and demonstrate how the resultant time-course calibrated model can be used as a powerful platform for metabolic engineering of *A. niger*.

## Results

### Citric acid fermentation occurs as part of a diauxic growth response

**Fig. 1:**
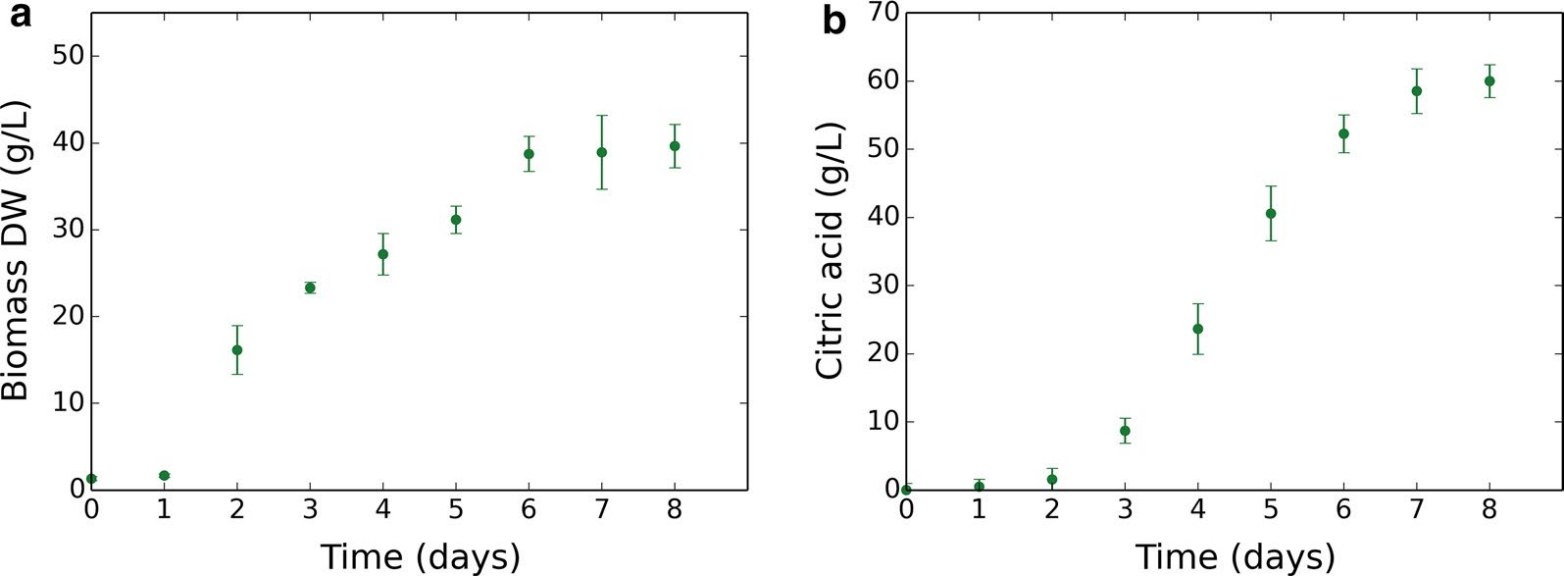
Citric acid production commences upon a diauxic growth switch. Empirical data plotted are the mean average of four biological replicates and error bars represent standard deviation. Citric acid data are normalised to reflect the amount produced. **a** Change in biomass dry weight (g/L) over time. **b** Change in external citric acid concentration (g/L) over time

To investigate citric acid production by the parent citric acid-producing ATCC 1015 strain, empirical time-course data were obtained from fermentation performed in shake flasks. Biomass and citric acid production were monitored with samples taken at 24-h time-points. Diauxic growth behaviour was observed, with a drop in growth rate at day 3 (Fig. 1a). Citric acid production commenced at day 3, coinciding with the diauxic growth shift (Fig. 1b). 60 g/L citric acid was produced.

**Fig. 2:**
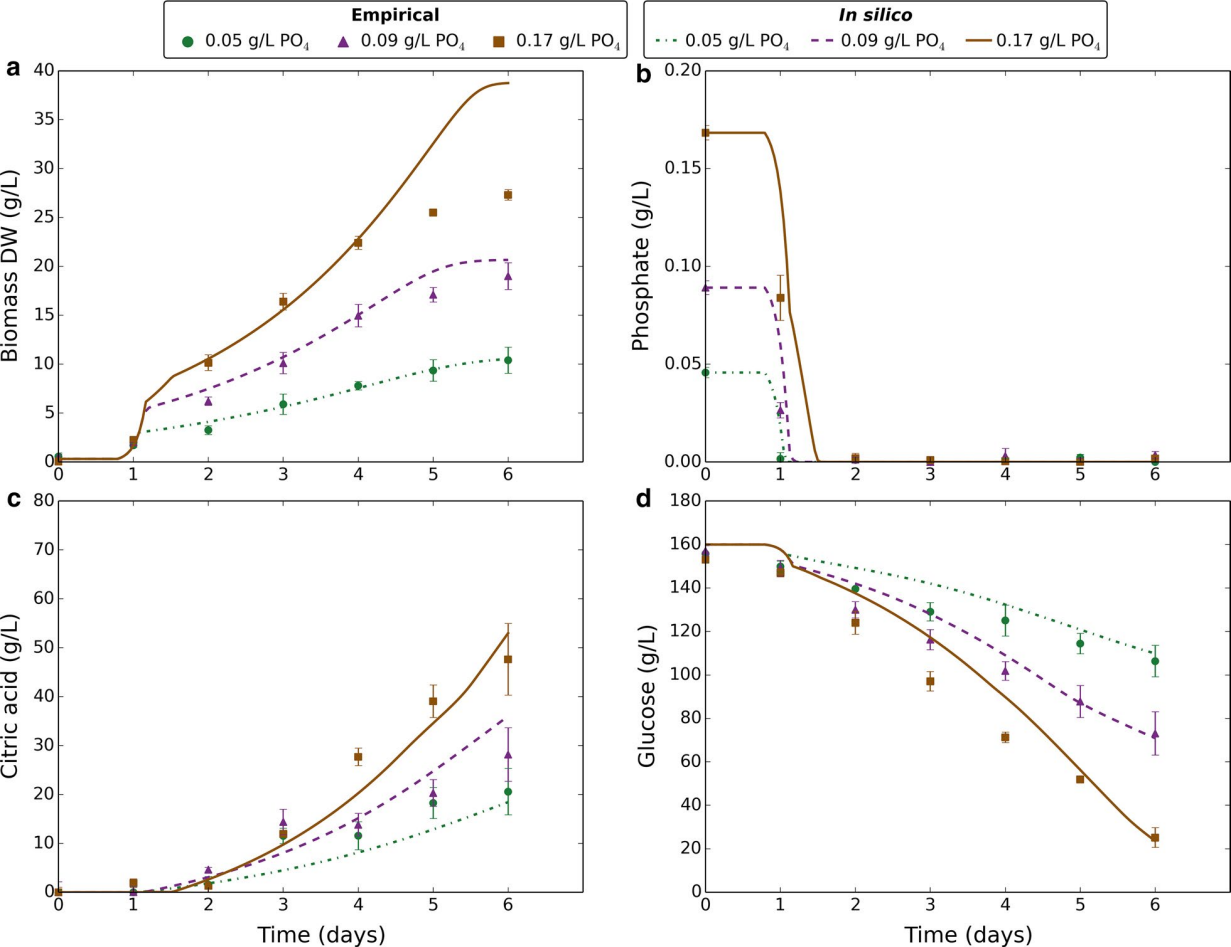
Comparing empirical and in silico data in response to varying phosphate. Markers represent empirical data and lines represent in silico data. Green circles and dashed-dotted lines correspond to 0.05 g/L phosphate. Purple triangles and dashed lines correspond to 0.09 g/L phosphate. Brown squares and solid lines correspond to 0.17 g/L phosphate. Empirical data plotted are the mean average of four biological replicates and error bars represent standard deviation. Citric acid data are normalised to reflect the amount produced. In silico data-points are one per minute. **a** Change in biomass dry weight (g/L) over time. **b** Change in external phosphate concentration (g/L) over time. **c** Change in external citric acid concentration (g/L) over time. **d** Change in external glucose concentration (g/L) over time

In order to better understand the basis of this growth behaviour, we developed a dynamic flux balance analysis (dFBA) model based on the previously published FBA model [13, 14]. To validate the model and further investigate the diauxic growth behaviour, empirical data were obtained for citric acid fermentation under a range of phosphate levels (0.05, 0.09, and 0.17 g/L). Samples of the cultures were taken every 24 h to produce a time-course of biomass dry weight, phosphate depletion, citric acid production, and glucose consumption (Fig. 2). Phosphate was rapidly taken up and depleted by day 2 (Fig. 2b), yet growth continued (Fig. 2a). Phosphate was therefore clearly stored internally to enable growth during the absence of external phosphate. Diauxic growth was observed, with growth becoming phosphate-limited. The diauxic growth shift was synchronous with depletion of external phosphate. The phosphate-limited growth rate was a function of the initial phosphate concentration, with increased growth rate at higher phosphate. The timing of citric acid production was observed to coincide with the onset of phosphate-limited growth and external phosphate depletion (Fig. 2c). Up to 50 g/L citric acid was produced, with the culture at 0.17 g/L phosphate producing the most. Glucose uptake was relatively slow for the lower phosphate cultures and a limiting factor in citric acid production (Fig. 2d).

From these observations, we hypothesised that the diauxic growth shift is caused by a switch to phosphate-limited growth, resulting in citric acid production. This hypothesis was motivated by examination of our data, existing knowledge of *A. niger* [10] and also the ecological evidence that organic acids are released extracellularly in order to facilitate the mobilisation of phosphate, especially in soil [15]. We decided to examine the plausibility of this hypothesis using dFBA modelling.

### Simulating citric acid fermentation by dynamic flux balance analysis

To create time-course simulations comparable to the citric acid fermentation empirical data, dynamic flux balance analysis (dFBA) was used with the *i*MA871 metabolic model [13]. Citric acid production was modelled by incorporating kinetic acid-dissociation reactions into the dFBA schema for the organic acids in *i*MA871 and setting the objective to proton production. This explicit inclusion leads to an acid hierarchy [14], which suggested that citric acid production was the most efficient means of acidification with oxalic acid production switched off.

**Fig. 3:**
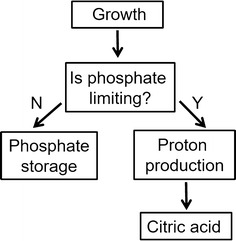
Simulating citric acid fermentation by dynamic flux balance analysis. A schematic showing the decision process implemented in the dFBA model

In the standard setting for the metabolic model, citric acid secretion is included as a part of the external constraints during growth [13]; however, this is not supported by our observations. Therefore, a novel modelling approach was designed to simulate the diauxic growth behaviour with citric acid production commencing upon a diauxic growth shift coupled to phosphate intake. To achieve this, a double optimisation dFBA setup was designed (Fig. 3). The objective is first set to biomass production, with the maximised growth rate then used in the second optimisation. The second objective is dependent on the growth-limiting condition of the first optimisation. The decision process uses a boolean expression. If the external phosphate flux is lower than its flux constraint, the second objective is set to phosphate storage to store excess phosphate not used for growth. Otherwise, external phosphate flux is equal to its flux constraint and the second objective is set to proton production to make use of the carbon not used for growth (phosphate-limited growth).

The dynamic modelling approach, dFBA, therefore includes a number of metabolite pools that are tracked outside of the FBA, including external glucose, external phosphate, external pH, organic acids as well as the hypothesised stored phosphate. These metabolite pools are linked to the FBA simulations at each step via first order differential equations describing transport processes. These differential equations are solved at each time-step to provide flux constraints for the FBA optimisations occurring in a tandem fashion and assuming the metabolic system remains at a steady-state despite the small changes in the external constraints. All equations used are detailed in the methods, but are essentially either linear diffusion or Michaelis–Menten transport equations across the membrane as described below and mathematically in the methods section. Literature sources were used to parameterise the model wherever available as described below.

Following previous studies [16, 17], glucose uptake was modelled as the sum of passive diffusion and facilitated diffusion, using empirical values from the literature [16, 17] for all transport-mediated kinetic parameters (Table 1). The calculated parameter for passive diffusion overestimated glucose uptake, and therefore was fitted to empirical data (Table 2). Transport-mediated glucose uptake in *A. niger* is inhibited by low pH and non-competitively inhibited by external citrate [17], and this was therefore included in the modelled glucose uptake. *A. niger* has both low- and high-affinity glucose transport systems [17], both of which were included in the model. The low-affinity system is reported only active above 150 g/L glucose, [17] and so this system was only included in the model at high glucose (> 150 g/L).

**Table 1:** Parameters set to empirical values from the literature

Parameter	Description	Value	References
*v*_*G*2,max_ (mmol gDW^−1^ h^−1^)	External glucose high-affinity transport maximum rate	0.186	[16, 17]
*K*_*G*2_ (mM)	External glucose high-affinity transport Michaelis constant	0.26	[16, 17]
*K*_*i*2_ (mM)	External glucose high-affinity transport citrate inhibition constant	933	[16, 17]
*v*_*G*3,max_ (mmol gDW^−1^ h^−1^)	External glucose low-affinity transport maximum rate	2.706	[16, 17]
*K*_*G*3_ (mM)	External glucose low-affinity transport Michaelis constant	3.67	[16, 17]
*K*_*i*3_ (mM)	External glucose low-affinity transport citrate inhibition constant	233.21	[16, 17]
*v*_GOX,max_ (mmol gDW^−1^ h^−1^)	Glucose oxidase (GOX) maximum reaction rate	27.48 × [GOX]^a^	[26]
*K*_GOX_ (mM)	Glucose oxidase (GOX) Michaelis constant	33	[26, 27]

^a^[GOX] is concentration of external glucose oxidase enzyme in mg gDW^−1^ and was fitted to empirical data (Table 2)

**Table 2:** Parameters fitted to our empirical data

Parameter	Description	Value
*v*_Pe,max_ (mmol gDW^−1^ h^−1^)	External phosphate maximum input rate^a^	0.08
K_Pe_ (mM)	External phosphate Michaelis constant	0.0333
*v*_*P*,max_ (mmol gDW^−1^ h^−1^)	Internal phosphate maximum input rate	0.0008
*K*_*P*_ (mM)	Internal phosphate Michaelis constant	0.0833
*v*_*G*1_ (mmol gDW^−1^ h^−1^)	External glucose passive uptake rate	0.00031419 × [GLC]^b^
*v*_*X*1_ (mmol gDW^−1^ h^−1^)	External xylose passive uptake rate	0.00033 × [XYL]^c^
*v*_*X*2,max_ (mmol gDW^−1^ h^−1^)	External xylose high-affinity transport maximum rate	0.2
*K*_*X*2_ (mM)	External xylose high-affinity transport Michaelis constant	3.33
*v*_*X*3,max_ (mmol gDW^−1^ h^−1^)	External xylose low-affinity transport maximum rate	2.5
*K*_*X*3_ (mM)	External xylose low-affinity transport Michaelis constant	3.33
[GOX] (mg gDW^−1^)	Concentration of external glucose oxidase enzyme	0.1
*v*_CIT_ (mmol gDW^−1^ h^−1^)	Citric acid output rate constraint^d^	0.12
*v*_OXAL_ (mmol gDW^−1^ h^−1^)	Oxalic acid output rate constraint	0.01

^a^External phosphate input rate changed 8 h after the dFBA start time to 0.015 mmol gDW^−1^ h^−1^ if initial pH 2 or 0.004 mmol gDW^−1^ h^−1^ if initial pH 7

^b^[GLC] is concentration of external glucose in mM

^c^[XYL] is concentration of external xylose in mM

^d^Citric acid output rate constraint changed to 0.016 mmol gDW^−1^ h^−1^ if initial pH above 2

Phosphate uptake and release of stored phosphate were modelled according to Michaelis–Menten kinetics. As no characterised phosphate transporters could be found for *A. niger* in the literature, kinetic parameters were fitted to empirical data on phosphate uptake (Table 2).

### Fitting of model parameters to empirical data and model validation

**Fig. 4:**
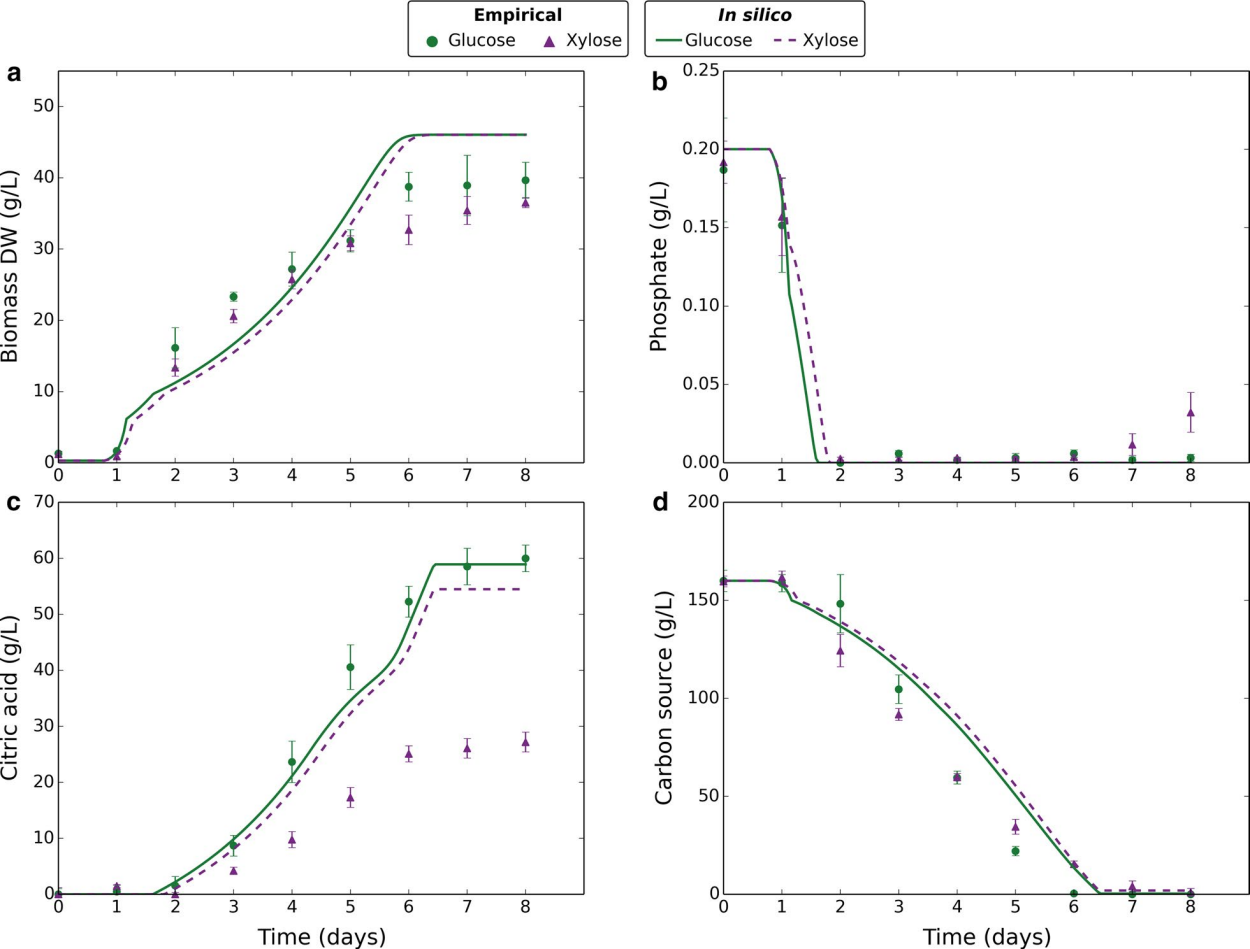
Comparing empirical and in silico data in response to different carbon sources. Markers represent empirical data and lines represent in silico data. Green circles and solid lines correspond to glucose. Purple triangles and dashed lines correspond to xylose. Empirical data plotted are the mean average of four biological replicates and error bars represent standard deviation. Citric acid data are normalised to reflect the amount produced. In silico data-points are one per minute. **a** Change in biomass dry weight (g/L) over time. **b** Change in external phosphate concentration (g/L) over time. **c** Change in external citric acid concentration (g/L) over time. **d** Change in external carbon source concentration (g/L) over time

The empirical data obtained from the experiment varying phosphate were used to fit model parameters and validate the model. A total of eight parameters were fitted to a data-set containing 84 data-points. As each data-point was in quadruplicate with very low error margins, we decided to use the data-set for both model training and validation. The trained model was later applied to independent data-sets (Fig. 4), which gave further validation. Using the trained model, citric acid fermentation was simulated for each of the phosphate levels tested, and model predictions plotted alongside empirical data (Fig. 2). The modelled diauxic growth behaviour gave good fits to empirical data, with external phosphate depletion being the trigger that results in phosphate-limited growth and citric acid production. All the model outputs showed a strong qualitative comparison to the empirical data with unfitted parameters taken directly from the literature. Notably, the modelled glucose uptake fitted empirical data closely (Fig. 2d) with unadjusted literature values for transport-mediated uptake rate and affinity.

However, a number of adjustments were required for the model to fit the empirical data more closely. In particular, the model underestimated biomass production during phosphate-limited growth, suggesting a lower phosphate demand not reflected in the *i*MA871 biomass equation. These contrasting observations in the different areas of growth suggest that the biomass equation for the *i*MA871 model represents an average biomass composition over different growth conditions and that therefore the biomass equation needs to be altered. Differences in biomass composition in different growth conditions have previously been reported in *Escherichia coli* [18]. To reflect citric acid-producing conditions, two new fitted parameters were added to the model, the nucleic acid and phospholipid components of the biomass equation (Additional file 1: Table S1). The ratios between the different components of each, and the total mass of the biomass components were kept constant. Change in mass was balanced by adjustment of the glycerol component, which has been reported to increase during citric acid-producing conditions [19]. The additional parameters increase the complexity of the model, and the likelihood of overfitting. Therefore, Akaike information criterion (AIC) [20] was used to measure the quality of fit and assess improvement in the model (Table 3) (see “Methods”).

**Table 3:** AIC scores for model selection

Additional parameters	Number of fitted parameters	AIC score
None	5	438
Nucleic acid component of biomass equation	6	416
Phospholipid component of biomass equation	6	422
Nucleic acid and phospholipid components of biomass equation	7	393
Nucleic acid and phospholipid components of biomass equation, and citric acid output constraint	8	300

Our model initially overestimated citric acid production. This may be due to the many internal constraints imposed on the internal metabolism by the intracellular accumulation of, or simply high throughputs of citrate that are not accounted for by the steady-state methodology of flux balance analysis. For example, the citrate sensitivity of 6-phosphofructo-1-kinase is a target of attempts to increase citrate production [21] and the rates of mitochondrial citrate export [22] and citrate secretion may be limiting. To reflect these constraints, a limit to the citric acid output rate, *v*_CIT_, was added and fitted as a parameter to more closely reflect empirical data (Table 2). Carbon uptake was decreased slightly as a result of the constraint on citric acid output, but still gave close fits to empirical data. The new model was assessed by calculating the AIC (see “Methods”), which showed a significant improvement (Table 3).

### Citric acid production on other carbon sources

To further investigate the diauxic growth behaviour, we tested citric acid fermentation using d-xylose as a substrate at an initial concentration of 160 g/L. The same diauxic growth shift coupled citric acid response was seen with xylose (Fig. 4) as seen with glucose. We applied our model, with previously fitted parameters unchanged. The empirical data from this experiment were not used in previous model training, and served to provide further validation with glucose as substrate and at a different phosphate level. The uptake rate of xylose was modelled similarly to glucose as the sum of passive and facilitated diffusion. The kinetic parameters for xylose uptake were fitted to our empirical data (Table 2). Close fits were achieved for biomass production and carbon source consumption, demonstrating the wide applicability of the dynamic model. Citric acid production was overestimated by the model, which may suggest a further limiting factor with xylose as the carbon source. The constraint applied to citric acid output rate, *v*_CIT_, was the same as for glucose (Table 2). The discrepancy may be due to differing morphology as we observed decreased biomass pellet sizes and higher viscosity in cultures grown on xylose.

### Investigating the role of phosphate during citric acid fermentation

**Fig. 5:**
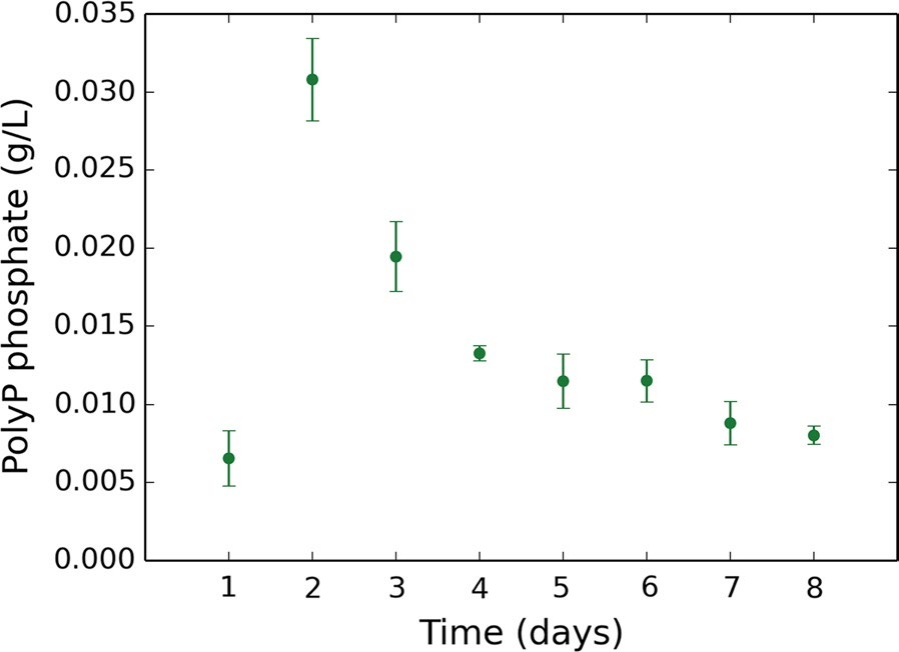
Change in polyphosphate levels during citric acid fermentation. Empirical data plotted are the mean average of 3 biological replicates and error bars represent standard deviation

As growth on glucose continued beyond external phosphate depletion (Fig. 2b), it became clear that *A. niger* has a phosphate storage mechanism, possibly via accumulation of polyphosphate as previously reported [23]. To investigate this, polyphosphate was extracted from biomass grown under citric acid-producing conditions and quantified. Polyphosphate levels were observed to rise early on in fermentation, peaking at day 2 at the point of external phosphate depletion (Fig. 5). Polyphosphate levels dropped rapidly from day 2 to day 4, with a more gradual decrease later in fermentation coinciding with phosphate-limited growth and citric acid production.

To further investigate the importance of phosphate, we searched for the genes encoding phosphate transporters in *A. niger* ATCC 1015. A total of eight putative genes were found (based on similarity to known transporters), suggesting that *A. niger* has evolved a range of phosphate uptake mechanisms as adaptation to different environmental conditions (Additional file 1: Table S2). It may be that only a subset of these genes encode phosphate transporters while others encode phosphate sensors. One of the genes (Accession Number EHA22558) has clear homologues in other species (Additional file 2: Figure S1), but none of these have been characterised or parameterised at the level of protein activity. The other gene annotations are more speculative so may not encode phosphate transporters [24].

### The dFBA model provides a platform for predictive metabolic engineering

A prediction of the model is that oxalic acid production is the most efficient means of acidification at initial pH 7, followed by citric acid. It is well known that *A. niger* predominantly secretes oxalic and gluconic acid at higher initial pH and that by imposing a low initial pH during fermentation, production of these competing organic acids is prevented and citric acid production is increased [14]. Our model suggests that by switching off oxalic acid production by deletion of oxaloacetate hydrolase (oah), citric acid will solely be produced. The model does not predict gluconic acid production suggesting that this may be decoupled from proton production and is instead a means of quickly sequestering glucose, through the action of extracellular glucose oxidase, early in fermentation.

**Fig. 6:**
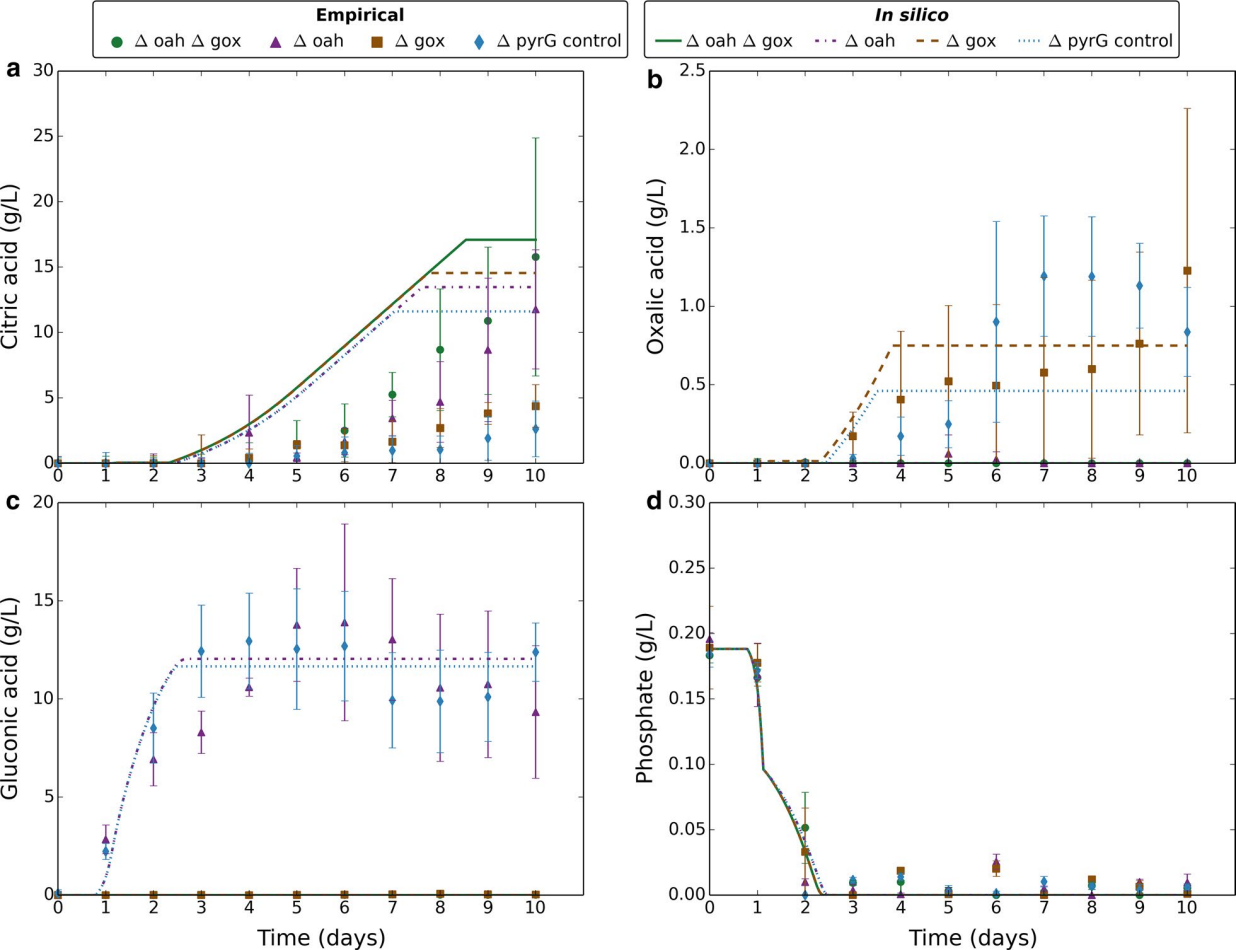
Comparing empirical and in silico data in response to Δoah and Δgox knockouts. Markers represent empirical data and lines represent in silico data. Green circles and solid lines correspond to Δoah Δgox. Purple triangles and dashed-dotted lines correspond to Δoah. Brown squares and dashed lines correspond to Δgox. Blue diamonds and dotted lines correspond to ΔpyrG control. Empirical data plotted is the mean average of four biological replicates and error bars represent standard deviation. Citric acid data are normalised to reflect the amount produced. In silico data-points are one per minute. **a** Change in external citric acid concentration (g/L) over time. **b** Change in external oxalic acid concentration (g/L) over time. **c** Change in external gluconic acid concentration (g/L) over time. **d** Change in external phosphate concentration (g/L) over time

To investigate this phenomenon, we engineered the ATCC 1015 strain by targeted gene deletion strategies to knockout oah and the gene encoding glucose oxidase (gox) responsible for gluconic acid production. We created two single knockouts (Δoah and Δgox) and a double knockout (Δoah Δgox), and characterised citric acid fermentation by these knockout strains at initial pH 7 (Fig. 6). Citric acid yield was significantly increased in the Δoah strain with a further marginal improvement in Δoah Δgox. This was not the case for the Δgox strain suggesting gluconic acid production occurs independently of proton production without impacting citric acid fermentation. Gluconic acid was produced early in fermentation while oxalic and citric acid production occurred later. The synchronicity of oxalic and citric acid production suggests that they are part of the same proton production response. In this experiment, the Mn^2+^ concentration was increased to 1000 ppb. Citric acid production usually requires Mn^2+^-deficient media, though was previously reported insensitive to Mn^2+^ in an oah and gox double negative mutant strain at pH 5 [25]. The presence of Mn^2+^ did not prevent citric acid production at initial pH 7, suggesting that its effect is limited to low pH conditions.

We applied our dFBA model to the Δoah and Δgox knockouts at initial pH 7, which gave close fits for oxalic and gluconic production. The differences in predicted citric acid production between the knockout strains showed a qualitative fit with empirical data (Fig. 6). However, constraints on oxalic and citric acid output rates, *v*_OXAL_ and *v*_CIT_, respectively (Table 2), were required to achieve the close fits. The constraint on citric acid output rate, *v*_CIT_, was different to that applied at initial pH 2. This may be due to morphological differences as we observed increased biomass pellet sizes when *A. niger* was grown at higher initial pH. The impact of differing morphology on transport processes and on anaerobicity within pellets requires further investigation. The widely reported absence of oxalic acid production below pH 2 [10, 25] was implemented in the model to reflect empirical data. To simulate gluconic acid production, the flux of the extracellular GOX and gluconic acid-dissociation reactions were forced depending on GOX kinetic parameters and the ambient pH. The kinetic parameters *V*_max_ and *K*_M_ of GOX (Table 1) were taken from the literature [26, 27]. The concentration of GOX per gram biomass dry weight is unknown for these experimental conditions so was fitted to empirical data (Table 2). The proportion of active GOX was based on empirical data of GOX activity at varying pH [28].

## Discussion

We have developed a novel dynamic model of *A. niger* citric acid fermentation that employs dFBA, to give time-course simulations of batch fermentation relevant to the industrial and experimental modes of *A. niger* fermentation. Our physiologically motivated double optimisation approach is a novel use of dFBA. Previous work incorporated proton production into the *i*MA871 metabolic model and used FBA in a static manner to give predictions on organic acid production [13, 14] at fixed values of pH. Since acid-dissociation reactions are dependent on the dynamic ambient pH, the application of dFBA with the dynamic tracking of pH enables more accurate predictions on organic acid production. The dynamic model was also expanded to include alternative feedstocks. Xylose was chosen as it is a pentose sugar abundant in hemicellulose in plant biomass and readily metabolised by *A. niger*. This new dynamic model is therefore a valuable addition to the *A. niger* metabolic modelling toolbox and a powerful demonstration of the promise of dFBA for applications in industrial biotechnology.

We tested the ability of the model to predict the impact of genetic modifications on organic acid fermentation at higher initial pH. We deleted genes encoding oxaloacetate acetylhydrolase (oah) and glucose oxidase (gox) to eliminate oxalic and gluconic production, respectively. Deletion of oah significantly increased citric acid production, and this was also observed in model predictions, though less pronounced. This suggests that the presence of oxalic acid in the cytosol in the oah positive strains may have negating effects on citric acid production not reflected by the model. It is expected that cytosolic organic acid accumulation may occur as a result of constrained transport, which is likely to have regulatory effects on organic acid production as a safeguard mechanism.

*Aspergillus niger* has been an industrial workhorse for decades and is essential to the world’s citric acid production. This is achieved through batch or fed-batch fermentation and the new model enables simulation of the dynamic process for the first time. The underlying causes of the naturally evolved property of organic acid production are still unclear. It was previously reported through static FBA predictions [14] that this may be driven by the biological objective of proton production. In line with empirical findings, oxalic acid production was revealed as the most efficient means of proton production at wide-ranging pH with citric acid second at low pH. We have now shown that this in a dynamic manner with variable external pH taken into account. Empirical data revealed that oxalic and citric acid production are synchronous upon a switch to phosphate-limited growth. This suggests that they are coupled and part of the same proton production response. This is further supported by the significant increase in citric acid production in Δoah.

The role of phosphate is striking as organic acid secretion has been reported in *A. niger* and other organisms as a phosphate mobilisation strategy [15, 29, 30]. The observed phosphate-limited growth results from the ability of *A. niger* to rapidly take up phosphate and store it as polyphosphate. The constraint on polyphosphate hydrolysis then limits growth, enabling flux of carbon to organic acid production. While *A. niger* has sufficient stored phosphate for growth, it does not use it and keeps it reserved. This behaviour may be due to the energy storage value of polyphosphate. We have observed a release of phosphate late in fermentation upon carbon depletion, which suggests that *A. niger* is capable of rapid polyphosphate hydrolysis as a means to create ATP when other energy sources are limiting. The control mechanisms that exist in *A. niger* to regulate polyphosphate hydrolysis and their relation to organic acid production warrant further investigation.

Our modelling approach has further demonstrated the potential of dFBA; the augmentation of static steady-state FBA by dynamic transport processes and time varying pools of metabolites. It has also revealed some fundamental issues with the application of these techniques to real applications. The objective function—the biomass equation—is fundamental to FBA and is typically constructed with evidence from mass spectrometry. Our work suggests that this function is strongly dependent on the fermentation context and may even be variable over the growth process. Biologically this is highly plausible, but dramatically increases the complexity of model implementation and fitting. In addition, it is clear that important regulatory constraints on the metabolic process, in this case citrate accumulation, need to be included. In this manner, we have created an augmented dFBA model in a potentially grey area between a complete kinetic model and the genetically based simplicity of an FBA model. Further work is required to fully understand validity of such models.

## Conclusions

Our findings reveal a naturally evolved behaviour that has been exploited by industry for decades to produce citric acid. Our work, encapsulated in a dynamic model, further elucidates the causative factors in organic acid fermentation by *A. niger* exploited by industrial processes. The model provides a means to further probe this behaviour and accurately explore the effects of genetic changes on organic acid fermentation in a dynamic manner. This new addition to the *A. niger* systems biology toolbox paves the way for metabolic engineering efforts to create new strains capable of enhanced citric acid production on low-cost feedstocks.

## Methods

### Shake flask experiments

Citric acid fermentation experiments were performed in 250-mL DeLong neck baffled shake flasks (Bellco Glass Inc.; Vineland, NJ, USA) with 30 mL medium. Flasks were siliconized with 2% (v/v) dimethyldichlorosilane. Cultures were incubated at 30 °C with shaking at 250 rpm. The following medium was used: glucose (160 g/L), urea (3.6 g/L), (NH_4_)_2_SO_4_ (0.52 g/L), K_2_HPO_4_ (0.5 g/L), CaCO_3_ (0.03125 g/L), MgSO_4_·7H_2_O (0.275 g/L), ZnSO_4_·7H_2_O (0.00225 g/L), FeSO_4_·7H_2_O (0.0095 g/L), CuSO_4_·5H_2_O (0.0117 g/L), MnCl_2_·(H_2_O)_4_ (0.0000108 g/L), citric acid monohydrate (3.3 g/L), Tween 80 (0.0094%). The Mn^2+^ concentration was confirmed as 7 ppb by ICP-MS (Biorenewables Development Centre, York, UK). The medium was autoclaved (121 °C 15 min) excluding glucose which was filter sterilised (0.22 µm). The pH of the medium was adjusted after autoclaving by the addition of sterile 2 M H_2_SO_4_. The medium included 10 mM uridine in experiments using pyrG negative strains. The medium was inoculated with 1 × 10^6^ spores/mL. Spores were harvested from potato dextrose agar slants incubated for 2 days at 37 °C. 2 mL saline Tween (0.1% Tween 80, 9 g/L NaCl) was added per slant and shaken to disperse spores. Spores were washed 3 times in saline Tween. 500 µL samples of cultures were taken every 24 h for determination of biomass, metabolites, and phosphate. Samples were collected in pre-dried, pre-weighed 1.5 mL Eppendorf tubes and centrifuged at 9000*g* for 5 min. The supernatant was retained for metabolite analysis and phosphate determination and stored at − 20 °C.

### Biomass dry weight determination

Mycelia were washed 4 times in 1 mL dH_2_O and centrifuged at 9000*g* for 5 min. Biomass was dried at 70 °C to constant weight. Biomass dry weight was determined by subtracting weight of the pre-dried 1.5 mL Eppendorf tube.

### Metabolite analysis

Enzymatic assay kits were used to determine the level of metabolites. Glucose, citric acid, xylose, glycerol, and gluconic acid were determined using Megazyme assay kits (K-GLUC, K-CITR, K-XYLOSE, K-GCROLGK, and K-GATE, respectively) (Megazyme International Ireland Ltd., Wicklow, Ireland). Oxalic acid was determined using the LIBIOS oxalate assay kit (Oxalate-100; LIBIOS, France).

### Phosphate determination

Phosphate was determined by the ammonium molybdate method, using an assay kit (ab65622; Abcam, Cambridge, UK).

### Polyphosphate extraction and quantification

Mycelia were grown up in shake flasks using the same method as previously described. Mycelia were harvested at 8 time-points (days 1–8) in triplicate. To obtain sufficient biomass, one flask was harvested per sample. Day 1 samples required the pooling of four flasks per replicate. Mycelia were harvested using a double layer of Miracloth (Calbiochem) and washed in 300 mL ice-cold 100 mM Tris·HCl pH 7 followed by 600 mL ice-cold dH_2_O. Washed mycelia were transferred to 15-mL Falcon tubes, flash frozen in liquid nitrogen, freeze dried, and stored at − 80 °C. Freeze dried mycelia were weighed out in 2 mL vials, approximately 50 mg per vial. Biomass was ground using the TissueLyser II (QIAGEN; Crawley, UK) at 30 Hz for 90 s 3 times. Each vial contained two beads. Powdered mycelia were lysed by adding 2 mL 10% (w/v) lysing enzymes from *Trichoderma harzianum* (Sigma, Dorset, UK) and incubating at 30 °C with shaking for 3 h. Samples were centrifuged and supernatant discarded. Polyphosphate was extracted following a previously described protocol [23]. All centrifuge steps were done at 13,000 rpm for 10 min at 4 °C and all shaking was done at 30 rpm. The polyphosphate fraction was dried in a Savant SPD131DDA SpeedVac Concentrator (Thermo Fisher Scientific). Polyphosphate was quantified by measuring free phosphate before and after acid hydrolysis using the previously described phosphate determination method. Acid hydrolysis was performed by adding 2 mL 0.5 M H_2_SO_4_ to the dry pellet and boiling at 100 °C for 3 h.

### Dynamic modelling of organic acid fermentation

Modelling was performed using the *i*MA871 metabolic model [13] as the model for the flux balance analysis. During this project, a more complete model of *A. niger* metabolism was published [31] but as this retains the core of *i*MA871 and is not specific to ATCC 1015, we have not adopted this model. The FBA calculations were performed using bespoke Java code which implements the GLPK toolkit (GNU). dFBA routines were written directly into the Java code with the differential equations solved by simple time-stepping (Euler method) with small values for the time-step. The ODEs (ordinary differential equations) were solved according to

1 $$C_{n + 1} = C_{n} + tf_{n} B_{n} ,$$

where *C*_*n*+1_ is the mmol of compound at time-point *n* + 1; *C*_*n*_ is the mmol of compound at time-point *n*; *t* is the time-step (1/60 h); *f*_*n*_ is the flux (mmol gDW^−1^ h^−1^) at time-point *n*; and *B*_*n*_ is the biomass (gDW) at time-point *n*. The flux constraints at time-point *n* + 1 were calculated by the following kinetic equations. External phosphate input (PIe <==>) was constrained according to

2 $$v_{\text{Pe}} = \frac{{v_{\text{Pe,max}} P_{\text{e}} }}{{K_{\text{Pe}} + P_{\text{e}} }},$$

where *v*_Pe_ is the external phosphate uptake rate (mmol gDW^−1^ h^−1^) and *P*_e_ is the external phosphate concentration (mM).

Internal phosphate input (PI <==>) was constrained according to

3 $$v_{P} = \frac{{v_{{P,{\rm max}}} P}}{{K_{P} + P}},$$

where *v*_*P*_ is the internal phosphate input rate (mmol gDW^−1^ h^−1^) and *P* is the concentration of stored phosphate (mM).

If external glucose was below 150 g/L, external glucose uptake (DGLCe <==> DGLC) was constrained according to

4 $$v_{G} = v_{G1} G + \frac{{v_{{G2,{ {\rm max} }}} G}}{{K_{G2} \left( {1 + \frac{C}{{K_{i2} }}} \right) + G\left( {1 + \frac{C}{{K_{i2} }}} \right)}},$$

where *v*_*G*_ is the external glucose uptake rate (mmol gDW^−1^ h^−1^); *G* is the external glucose concentration (mM); and *C* is the external citrate concentration (mM).

If external glucose was greater than or equal to 150 g/L, external glucose uptake was constrained according to

5 $$v_{G} = v_{G1} G + \frac{{v_{{G2,{{\rm max} }}} G}}{{K_{G2} \left( {1 + \frac{C}{{K_{i2} }}} \right) + G\left( {1 + \frac{C}{{K_{i2} }}} \right)}} + \frac{{v_{{G3,{ {\rm max} }}} G}}{{K_{G3} \left( {1 + \frac{C}{{K_{i3} }}} \right) + G\left( {1 + \frac{C}{{K_{i3} }}} \right)}},$$

where *v*_*G*_ is the external glucose uptake rate (mmol gDW^−1^ h^−1^); *G* is the external glucose concentration (mM); and *C* is the external citrate concentration (mM).

If external xylose was below 150 g/L, external xylose uptake (XYLe <==>) was constrained according to

6 $$v_{X} = v_{X1} X + \frac{{v_{{X2,{ {\rm max} }}} X}}{{K_{X2} + X}},$$

where *v*_*X*_ is the external xylose uptake rate (mmol gDW^−1^ h^−1^), and *X* is the external xylose concentration (mM).

If external xylose was greater than or equal to 150 g/L, external xylose uptake was constrained according to

7 $$v_{X} = v_{X1} X + \frac{{v_{{X2,{ {\rm max} }}} X}}{{K_{X2} + X}} + \frac{{v_{{X3,{ {\rm max} }}} X}}{{K_{X3} + X}},$$

where *v*_*X*_ is the external xylose uptake rate (mmol gDW^−1^ h^−1^), and *X* is the external xylose concentration (mM).

The extracellular GOX (glucose oxidase) reaction rate was calculated according to

8 $$v_{\text{GOX}} = p_{\text{GOX}} \frac{{v_{\text{GOX,max}} G}}{{K_{\text{GOX}} + G}},$$

where *v*_GOX_ is the GOX reaction rate; *p*_GOX_ is the proportion of active GOX; and *G* is the external glucose concentration (mM).

The proportion of active GOX, *p*_GOX_, as a function of pH was determined according to

9 $$p_{\text{GOX}} = - 0.102{\text{pH}}^{2} + 1.082{\text{pH}} - 1.95.$$

The kinetic parameters were either fitted to our empirical data (Table 2) or set to empirical values from the literature if available (Table 1).

The *i*MA871 model was adapted to include proton production as an objective function and acid-dissociation reactions for seven acids (citric, oxalic, gluconic, acetic, malic, succinic, lactic) but as a function of a dynamic external pH rather than a fixed pH [14]. The number of protons released in each acid-dissociation reaction was calculated at each time-step according to the following equation based on ambient pH and pKa values.

10 $$H = \frac{{K_{1} \left( {H_{e} } \right)^{ - 1} + 2K_{1} K_{2} \left( {H_{e} } \right)^{ - 2} + 3K_{1} K_{2} K_{3} \left( {H_{e} } \right)^{ - 3} }}{{1 + K_{1} \left( {H_{e} } \right)^{ - 1} + K_{1} K_{2} \left( {H_{e} } \right)^{ - 2} + K_{1} K_{2} K_{3} \left( {H_{e} } \right)^{ - 3} }} ,$$

where *K*_1_, *K*_2_, and *K*_3_ are constants calculated from pKa values of each acid species (Table 4), and *H*_*e*_ is the external molar concentration of protons that is tracked in the dFBA as a dynamic pool.

**Table 4:** Acid constants for Eq. 10

Acid species	*K*_1_	*K*_2_	*K*_3_
Citric acid	10^−3.128^	10^−4.761^	10^−6.396^
Gluconic acid	10^−3.7^	0	0
Acetic acid	10^−4.757^	0	0
Malic acid	10^−3.459^	10^−5.097^	0
Succinic acid	10^−4.207^	10^−5.636^	0
Lactic acid	10^−3.86^	0	0
Oxalic acid	10^−1.252^	10^−4.266^	0

An output reaction was added for external protons (Hpe <==>), which was set as the objective when maximising proton production. An explicit phosphate storage reaction was also included in the dFBA. An input reaction for internal phosphate (PI <==>) was added to the metabolic model, and the dynamic pool of internal phosphate was tracked in the dFBA. This new reaction was set as the objective when maximising phosphate storage.

When plotting alongside empirical data, the dFBA start time was taken as the spore germination time, 18 h after inoculation. The initial biomass dry weight was set to 0.3125 g/L following empirical data.

### Model parameterisation

Glucose transport-mediated uptake [16, 17] and glucose oxidase [26, 27] kinetic parameters were calculated from empirical data in the literature (Table 1). The concentration of active GOX enzyme [GOX] was fitted to empirical data (Table 2). The other kinetic parameters in the model were fitted to empirical data via a manual fitting routine (Table 2).

### Quality of fit assessment and model selection

Akaike information criterion (AIC) [20] was used to measure the quality of fit and assess improvement in the model. The AIC was calculated according to

11 $${\text{AIC}} = 2k + n\;{ \ln }\left( {\frac{\text{RSS}}{n}} \right),$$

where *k* is the number of fitted parameters; *n* is the number of data-points; and RSS is the residual sum of squares.

### Targeted gene deletion of oah and gox

Targeted gene deletion was performed using a previously reported strategy [32]. As this technique requires a pyrG negative strain, the pyrG gene first had to be deleted from ATCC 1015. This was achieved using homologous recombination. ATCC 1015 was transformed with linear DNA containing 2 kb up- and 1.5 kb down-stream flanking regions of the pyrG gene (Accession Number EHA25155), kindly given by Kokolski (University of Nottingham). Polyethylene glycol (PEG)-mediated transformation of protoplasts was used [32]. Successful deletions were selected by resistance to 5-fluoroorotic acid (5-FOA) (Fluorochem; Derbyshire, UK) and uridine auxotrophy, and confirmed by PCR and DNA sequencing using primers external to the gene (pyrG_ex_fw and pyrG_ex_rv). The oah and gox genes were identified in the ATCC 1015 genome as Accession Numbers EHA22250 and EHA27180, respectively. 1.5 kb up- and down-stream flanking regions were cloned from ATCC 1015 gDNA using Phusion HF DNA polymerase (Thermo Fisher Scientific), and the following primers: oah_up_fw, oah_up_rv, oah_down_fw, oah_down_rv, gox_up_fw, gox_up_rv, gox_down_fw, gox_down_rv. 15-bp tails (underlined) were added to outermost primers for In-Fusion^®^ HD cloning (Clontech; France) into the pc3 vector between the *Not*I and *Spe*I restriction sites. To join up- and down-stream fragments together, overlap extension PCR was used with 30-bp overlapping tails (underlined) added to innermost primers. Overlapping fragments were first annealed as follows: 50 µL reaction containing 200 ng each fragment, 400 µM dNTPs, HF buffer, and 1 U Phusion HF DNA polymerase run on SOE1 programme (94 °C 5 min, then 94 °C 30 s, 60 °C 90 s, 72 °C 90 s 10 times, then 10 °C forever). The annealed product was then amplified using outermost primers as follows: 100 µL reaction containing 50 µL first reaction, 1 µM each primer, 400 µM dNTPs, HF buffer and 1 U Phusion HF DNA polymerase run on SOE2 programme (94 °C 2 min, then 94 °C 30 s, 60 °C 30 s, 72 °C 90 s 35 times, then 72 °C 10 min, 10 °C forever). The annealed product was gel purified using the QIAquick gel extraction kit (QIAGEN; Crawley, UK). Transformation was performed using XL10-Gold Ultracompetent cells according to the manufacturer’s instructions (Agilent Technologies; Cheshire, UK). Plasmid was isolated using the Wizard^®^ Plus SV minipreps DNA purification kit (Promega; Southampton, UK). Plasmid integrity was confirmed by DNA sequencing. ATCC 1015 ΔpyrG was transformed with the pc3-oah and pc3-gox deletion vectors using the previously reported PEG-mediated protoplast transformation protocol [32]. The gene deletion procedure previously outlined [32] was then followed with minor modifications. 1.5 g/L 5-FOA was used to select for pyrG negative colonies with incubation at 37 °C for 3 days. oah and gox knockouts were identified by PCR screening with primers external and internal to the deletion site (oah_ex_fw, oah_ex_rv, oah_int_fw, oah_int_rv, gox_ex_fw, gox_ev_rv, gox_int_fw, gox_int_rv). Gene deletion was further confirmed by DNA sequencing of the region external to the deletion site. To create the Δoah Δgox double knockout, the deletion procedure for gox was applied to ATCC 1015 ΔpyrG Δoah (Table 5).

**Table 5:** Primers used in this work

Primer	Nucleotide sequence (5′ to 3′)
pyrG_ex_fw	CTTTGCAGGTGTGGCTGAAC
pyrG_ex_rv	ACAGCAGTGCTTATCTGCGA
oah_up_fw	ACCGCGGTGGCGGCCGCGCTGTGTCCATACCATCAATCC
oah_up_rv	GAATGTTGCAGACAGACAGAAAGCAAAAGAGCAGGCAGTAGTAAGCAAGAAT
oah_down_fw	TCTTTCTTATTCTTGCTTACTACTGCCTGCTCTTTTGCTTTCTGTCTGTCTGC
oah_down_rv	CGGGGGATCCACTAGTTCTCCTCTTCCCCTGCCTTT
gox_up_fw	ACCGCGGTGGCGGCCGCGAGATGGCAATTTCCGCGAC
gox_up_rv	GAATATTCGAGGATTGTGGGAGAGACAGCGCGTGCAAACTCACCACCAAG
gox_down_fw	CTGTCTTGACCTTGGTGGTGAGTTTGCACGCGCTGTCTCTCCCACAATCC
gox_down_rv	CGGGGGATCCACTAGTCTACGCTCATGTCCTGGTCC
oah_ex_fw	TAAGGCTACCCAACCCACCC
oah_ex_rv	GCTTATCTAGGCCCCTGCTG
oah_int_fw	ACCCAACCACACCATCCTTC
oah_int_rv	ACCCAGTTCCCCACTAACAC
gox_ex_fw	CACTATCGCCAAGCAGGGAT
gox_ex_rv	AAGGTCTCGTTGAAGGTGGC
gox_int_fw	AGCAACCAGCCTTTCCTCTC
gox_int_rv	CCCAGTTCCAGCCCTCATTT

## Additional files

**Additional file 1: Table S1.** Biomass equation parameters altered to fit empirical data. **Table S2.** Putative phosphate transporters in ATCC 1015. Top BLASTP hits with phosphate transporters in SwissProt database are given.

**Additional file 2: Figure S1.** Phylogenetic tree of putative phosphate transporters in ATCC 1015.
