# Supplementary material for: An accurate description of Aspergillus niger organic acid batch fermentation through dynamic metabolic modelling
Source: Biotechnol Biofuels. 2017 Nov 9;10:258. doi: 10.1186/s13068-017-0950-6 (PMC5679502; doi:10.1186/s13068-017-0950-6)
Supplement: Supplementary file 1 — Additional file 1: Table S1. Biomass equation parameters altered to fit empirical data. Table S2. Putative phosphate transporters in ATCC 1015. Top BLASTP hits with phosphate transporters in SwissProt database are given. [file 13068_2017_950_MOESM1_ESM.docx]

**Table S1.** Biomass equation parameters altered to fit empirical data.

| **Compound** | **Before fitting (mmol gDW^-1^ h^-1^)** | **After fitting (mmol gDW^-1^ h^-1^)** |
| --- | --- | --- |
| AMP | -0.01402222 | -0.0046740733 |
| GMP | -0.01688834 | -0.0056294467 |
| CMP | -0.01402222 | -0.0046740733 |
| UMP | -0.01117424 | -0.0037247467 |
| DAMP | -0.00193736 | -0.0006457867 |
| DCMP | -0.00201544 | -0.0006718133 |
| DTMP | -0.00193736 | -0.0006457867 |
| DGMP | -0.00201544 | -0.0006718133 |
| PC | -0.015312 | -0.005104 |
| PS | -0.000359 | -0.0001196667 |
| PE | -0.034807 | -0.0116023333 |
| GL | -0.46 | -0.9030928715 |
| ADP | 71.60986992 | 71.5025795733 |
| PI | 71.60986992 | 71.5025795733 |
| ATP | -71.60986992 | -71.5025735267 |
| H2O | -69.08036756 | -69.0511151733 |

**Table S2.** Putative phosphate transporters in ATCC 1015. Top BLASTP hits with phosphate transporters in SwissProt database are given.

| **ATCC 1015 locus tag** | **GenBank accession** | **Top BLASTP hit (SwissProt)** | **Identity (%)** | **E-value** |
| --- | --- | --- | --- | --- |
| ASPNIDRAFT_173247 | EHA22558 | P15710.1 | 37 | 4e-130 |
| ASPNIDRAFT_190334 | EHA20653 | P25297.2 | 34 | 2e-90 |
| ASPNIDRAFT_121846 | EHA27663 | Q7RVX9.2 | 61 | 0.0 |
| ASPNIDRAFT_52154 | EHA22720 | O42885.2 | 29 | 2e-42 |
| ASPNIDRAFT_42307 | EHA25335 | Q9S735.1 | 27 | 6e-12 |
| ASPNIDRAFT_175394 | EHA23128 | Q8H074.1 | 25 | 2e-26 |
| ASPNIDRAFT_206238 | EHA26306 | P27514.2 | 41 | 0.0 |
| ASPNIDRAFT_35379 | EHA27197 | Q8H074.1 | 24 | 2e-22 |
