# Supplementary figures and images for: An accurate description of Aspergillus niger organic acid batch fermentation through dynamic metabolic modelling
Source: Biotechnol Biofuels. 2017 Nov 9;10:258. doi: 10.1186/s13068-017-0950-6 (PMC5679502; doi:10.1186/s13068-017-0950-6)

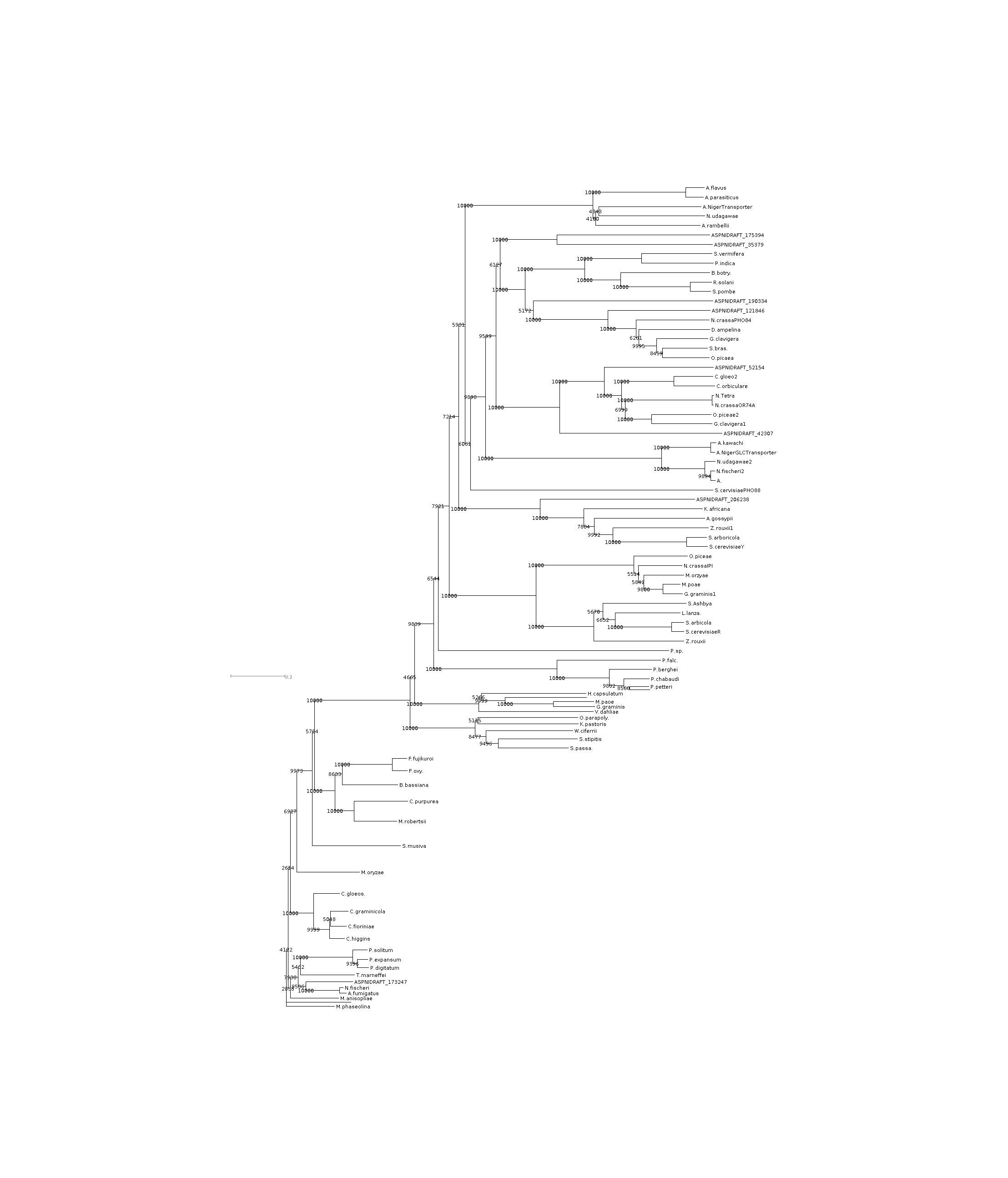

Supplement: Supplementary file 2 — Additional file 2: Figure S1. Phylogenetic tree of putative phosphate transporters in ATCC 1015. [file 13068_2017_950_MOESM2_ESM.tiff]
